# Supplementary material for: Brain Networks Sensitive to Object Novelty, Value, and Their Combination
Source: Cereb Cortex Commun. 2020 Jul 29;1(1):tgaa034. doi: 10.1093/texcom/tgaa034 (PMC7503454; doi:10.1093/texcom/tgaa034)
Supplement: Supplemental_Figures_revision_v05_sml_tgaa034 [file supplemental_figures_revision_v05_sml_tgaa034.pdf]

Supplemental Figures

Brain networks sensitive to object novelty, value, and their combination

Ali Ghazizadeh*[1,2], Mohammad Amin Fakharian[1], Arash Amini[1], Whitney Griggs[3], David A. Leopold[4,5], Okihide Hikosaka[3,6]

[1]Bio-intelligence Research Unit, Electrical Engineering Department, Sharif University of Technology, Tehran 11365-11155, Iran

[2]School of Cognitive Sciences, Institute for Research in Fundamental Sciences, Tehran 19395-5746, Iran

[3]Laboratory of Sensorimotor Research, National Eye Institute, NIH, Bethesda, MD 20892, USA

[4]Laboratory of Neuropsychology, National Institute of Mental Health, National Institutes of Health, Bethesda, MD 20892;

[5]Neurophysiology Imaging Facility, National Institute of Mental Health, National Institute of Neurological Disorders and Stroke, National Eye Institute, National Institutes of Health, Bethesda, MD 20892

[6]National Institute on Drug Abuse, NIH, Baltimore, MD 21224, USA

* corresponding author, Email: ghazizadeh@sharif.edu

Supp Fig 1

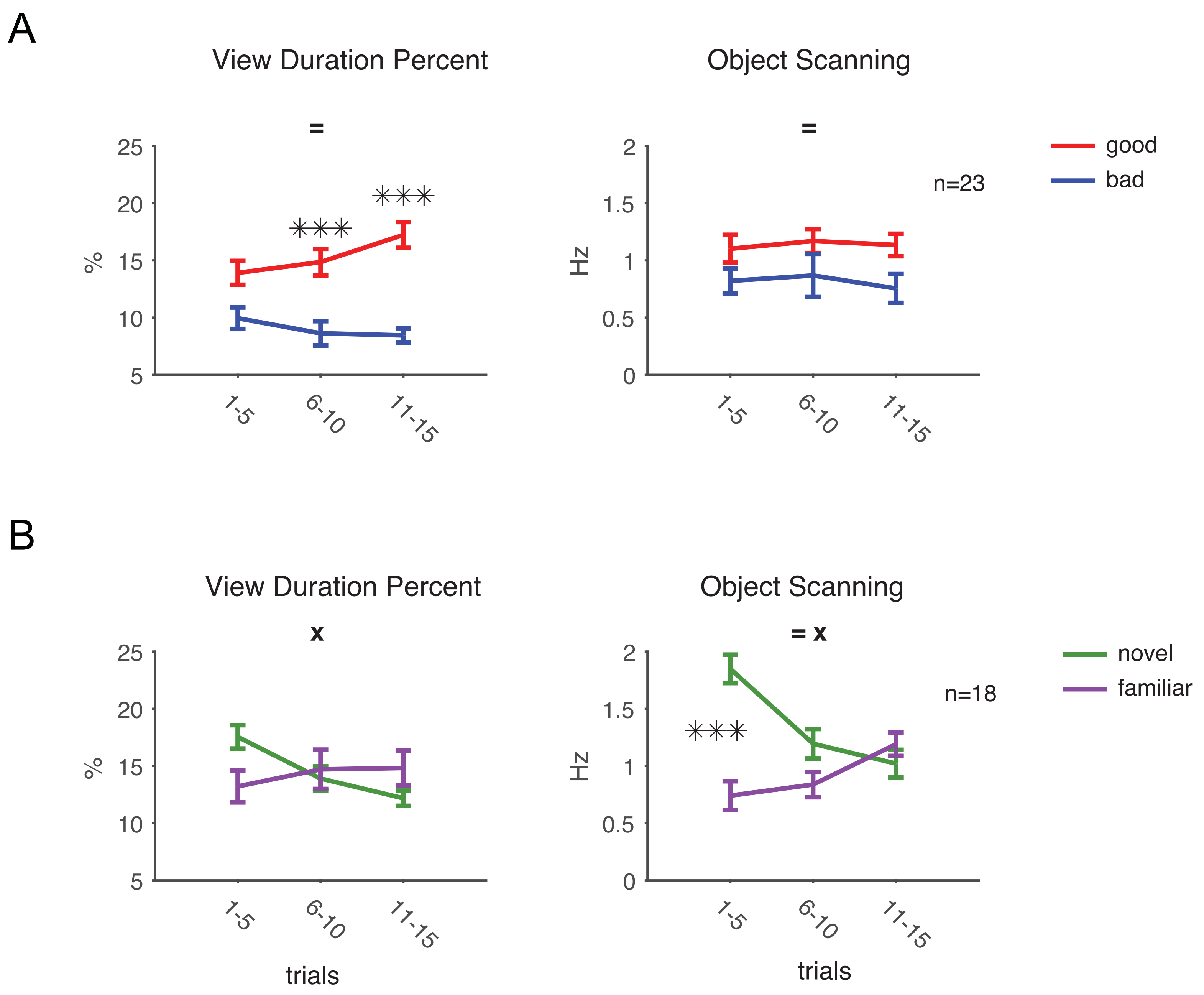

Supplementary Figure 1: Sustained gaze bias for value dimension and transient gaze bias for novelty dimension. Objects were randomly selected and shown to the monkey for viewing in the absence of reward (GB and NF in separate blocks of free viewing) A, Gaze bias for viewing good vs bad object was measured during each session of free viewing and averaged separately for early (trials:1-5), mid (trials:6-10) and late (trials:11-15) epochs. (= significant main effect of value or novelty, \ significant main effect of trial, x significant interaction). Post-hoc tests (hsd) are marked with asterisks. Gaze bias measures shown are viewing duration as a percentage of total trial duration and the frequency of object scanning saccades while viewing the object. ($F_{1,132}>9.35$, $p<0.002$ for main effect of value, $F_{2,132}<0.68$, $p>0.5$ for main effect of trials, $F_{2,132}<2.86$, $p>0.06$ interaction) B, same format as A but for novel vs familiar gaze bias. ($F_{2,102}>3.99$, $p<0.02$ interaction and for object scanning $F_{1,102}=19.7$, $p<1e-3$ main effect of novelty)

Supp Fig 2

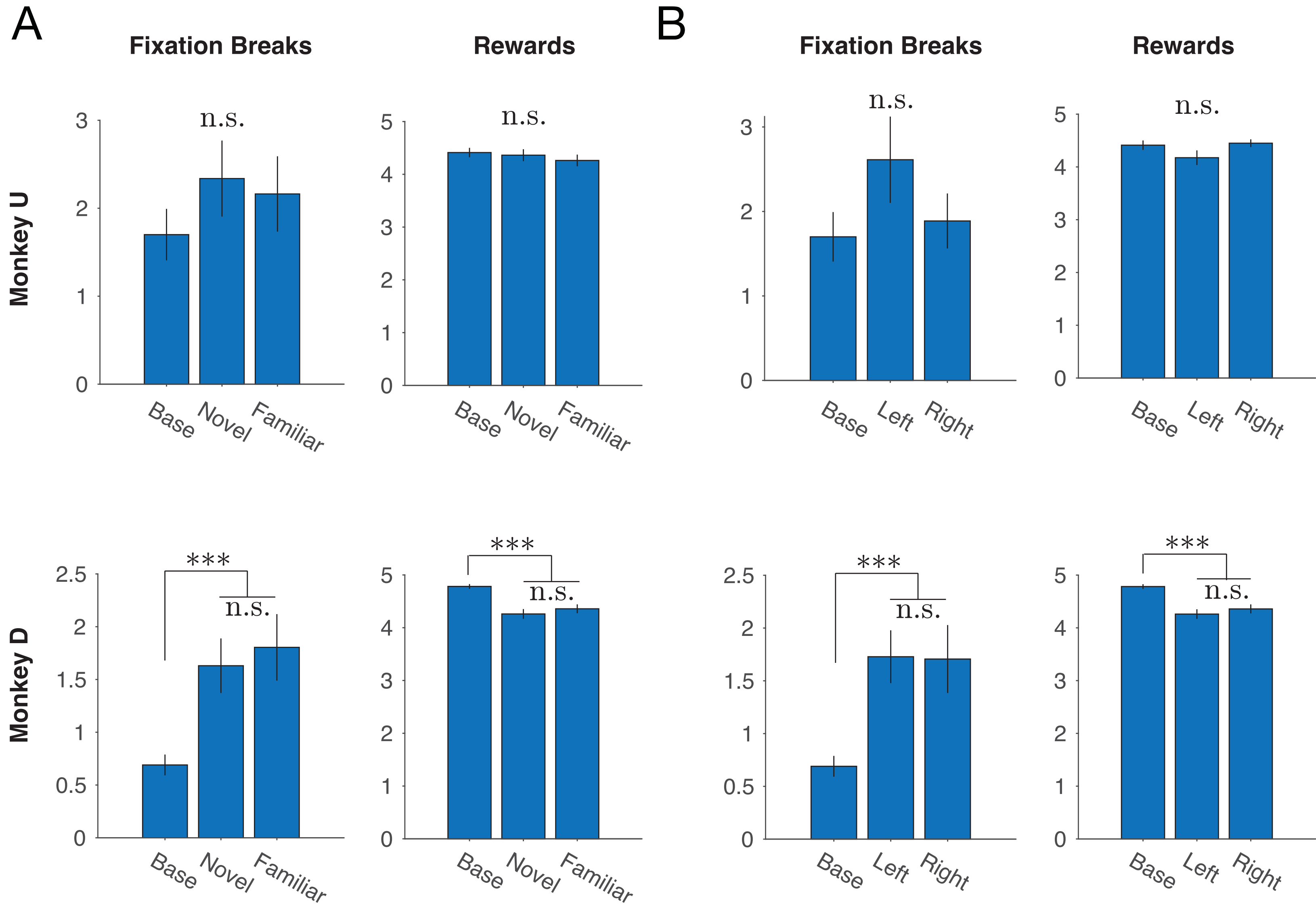

Supplementary Figure 2: Equivalent performance and rewards across blocks during NF scans. A, Number of fixation breaks (left) and number of rewards received (right) in the base, novel and familiar presentation blocks for each monkey. (monkey U F2,317<0.89, p>0.41 and monkey D F2,317>10.4, p<1e-4, but difference between novel and familiar blocks not significant hsd post-hoc p>0.6) B, same format as in A but for the base, left, and right presentation blocks. (monkey U F2,317<1.69, p>0.18 and monkey D F2,317>10.3, p<1e-4, but difference between novel and familiar blocks not significant hsd post-hoc p>0.6)

Supp Fig 3

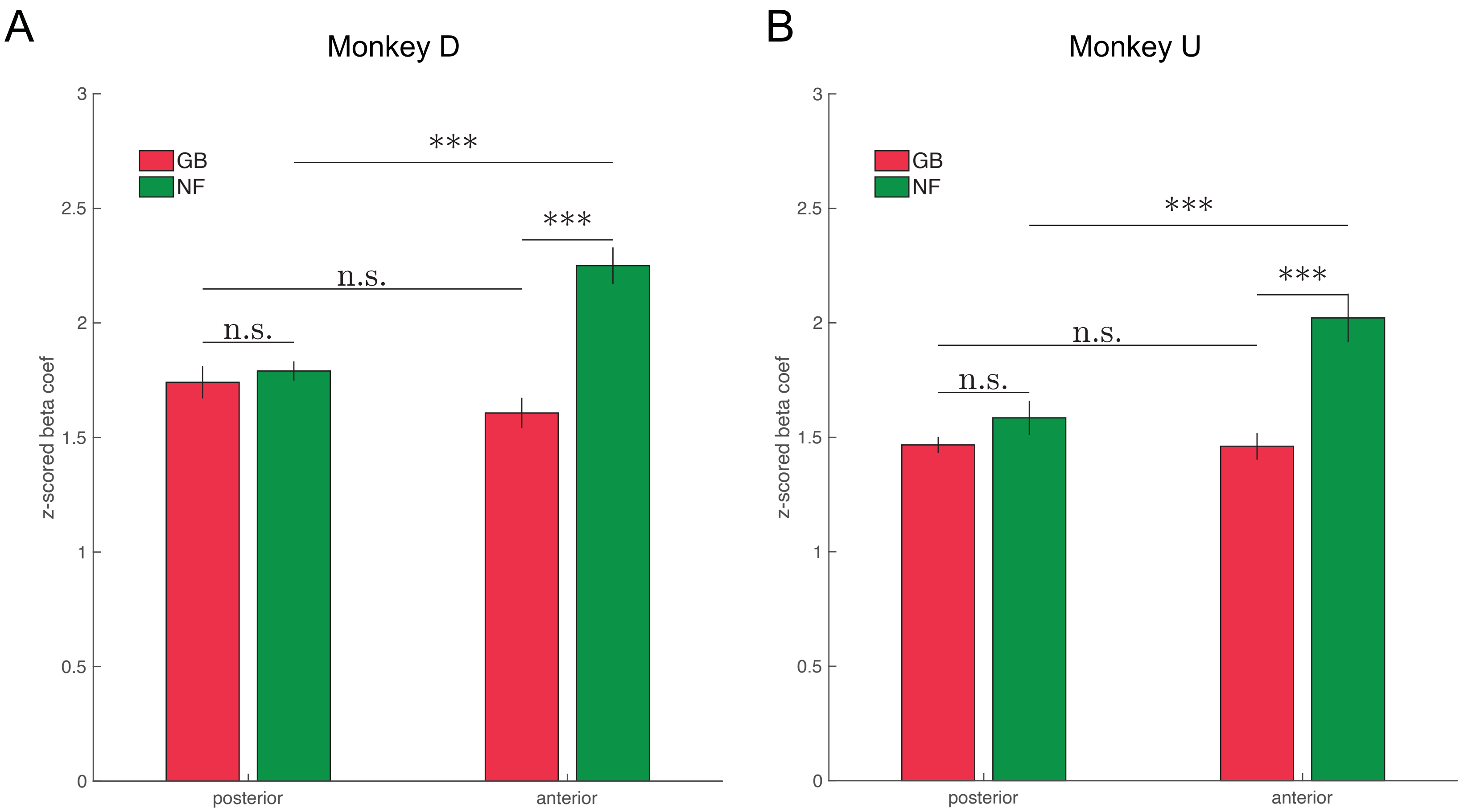

Supplementary Figure 3: Difference of NF and GB coding in the anterior and posterior parts of vSTS. A, NF and GB beta coefficients z-scored using all gray matter beta coefficients to render the two dimensions comparable for monkey D. B, Same as A for monkey U. Anterior vSTS included TEm/a and posterior vSTS included TEO,FST, IPa ,V4 and MT. Monkey U: $F_{3,522} = 10.37$, $p = 1e-6$, Monkey D: $F_{3,771} = 10.50$, $p = 3e-8$. Post-hoc test hsd ***$P<0.001$.

Supp Fig 4

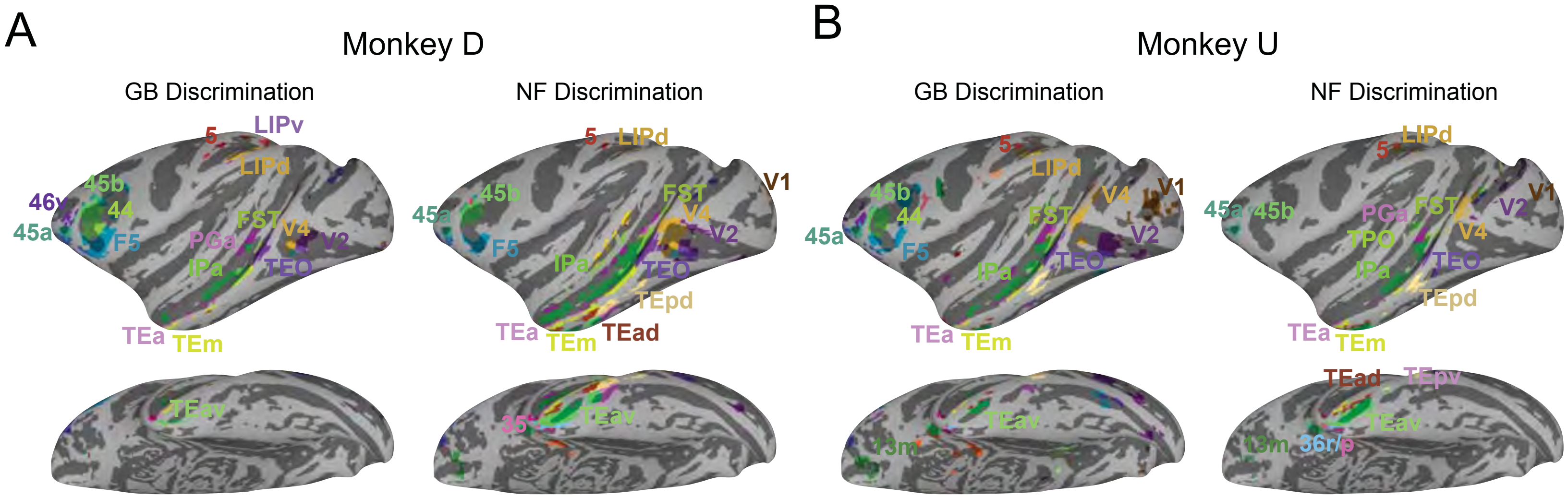

Supplementary Figure 4: Annotation of cortical areas with significant GB and NF coding A, Cortical areas corresponding to areas shown in Figure 2 in monkey D. B, Same format as A for monkey U.

Supp Fig 5

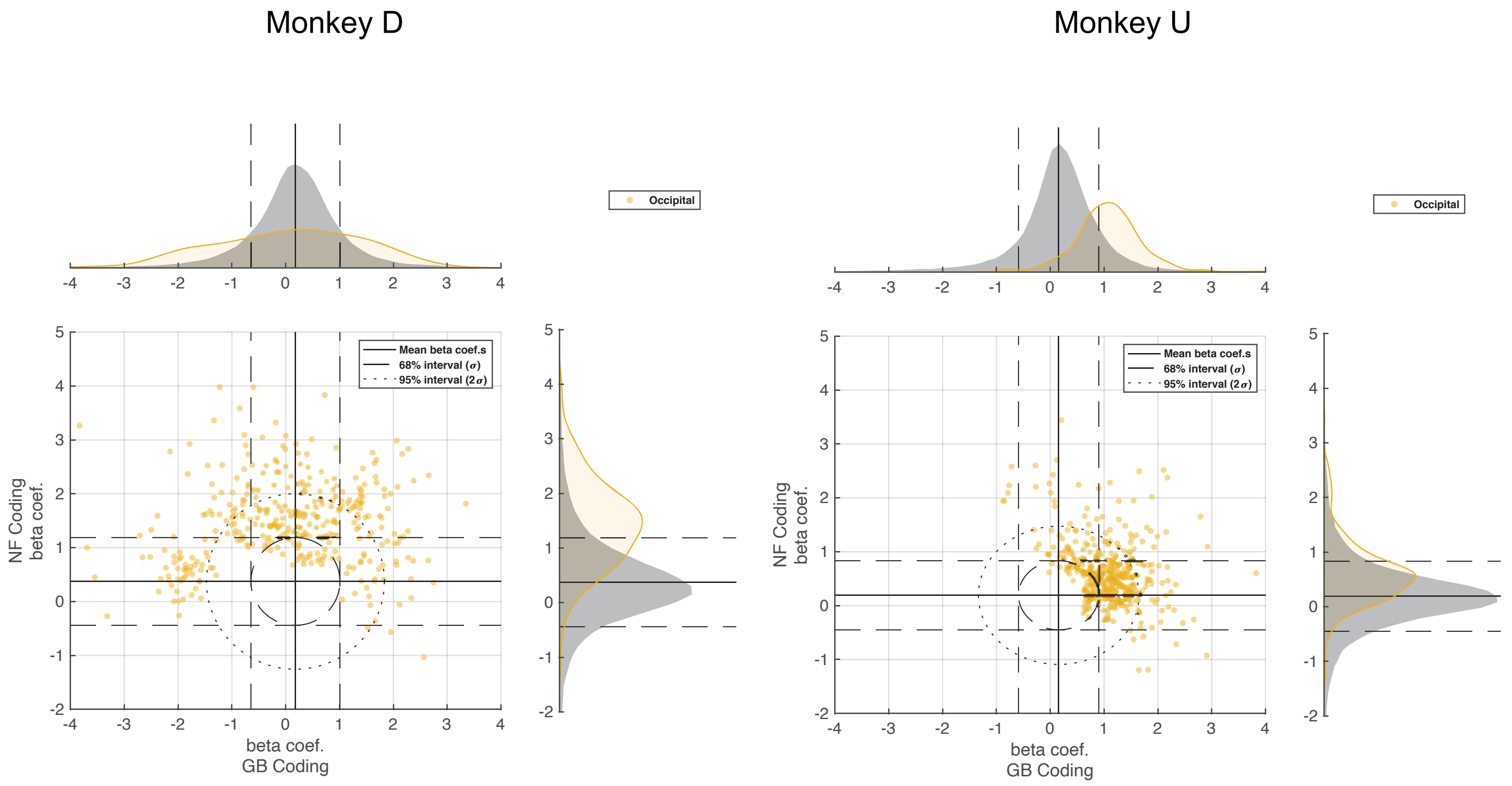

Supplementary Figure 5: Novelty and value coding in the occipital network. Same as Figure 3B,D but for occipital network for both monkeys. All voxels are plotted without regards to the 68% and 95% thresholds.

Supp Fig 6

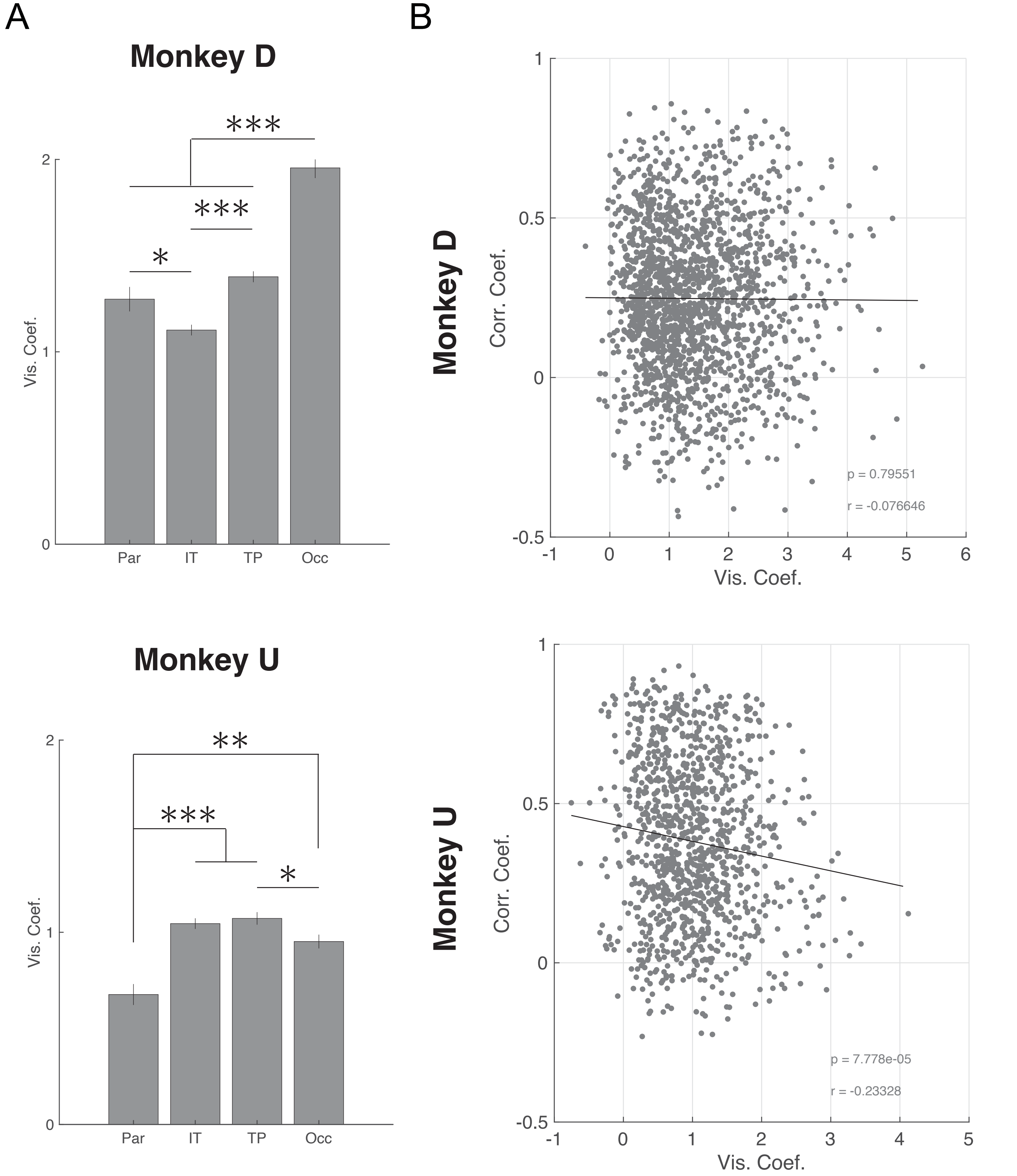

Supplementary Figure 6: Visual activation vs NF/GB coding across cortical networks. A, The average visual coefficients across networks for each monkey. B, The relationship between the NF-GB time-series correlation and visual activation of all voxels in all networks (each point represents a voxel). r indicates Pearson's correlation and the regression line is shown.

Supp Fig 7

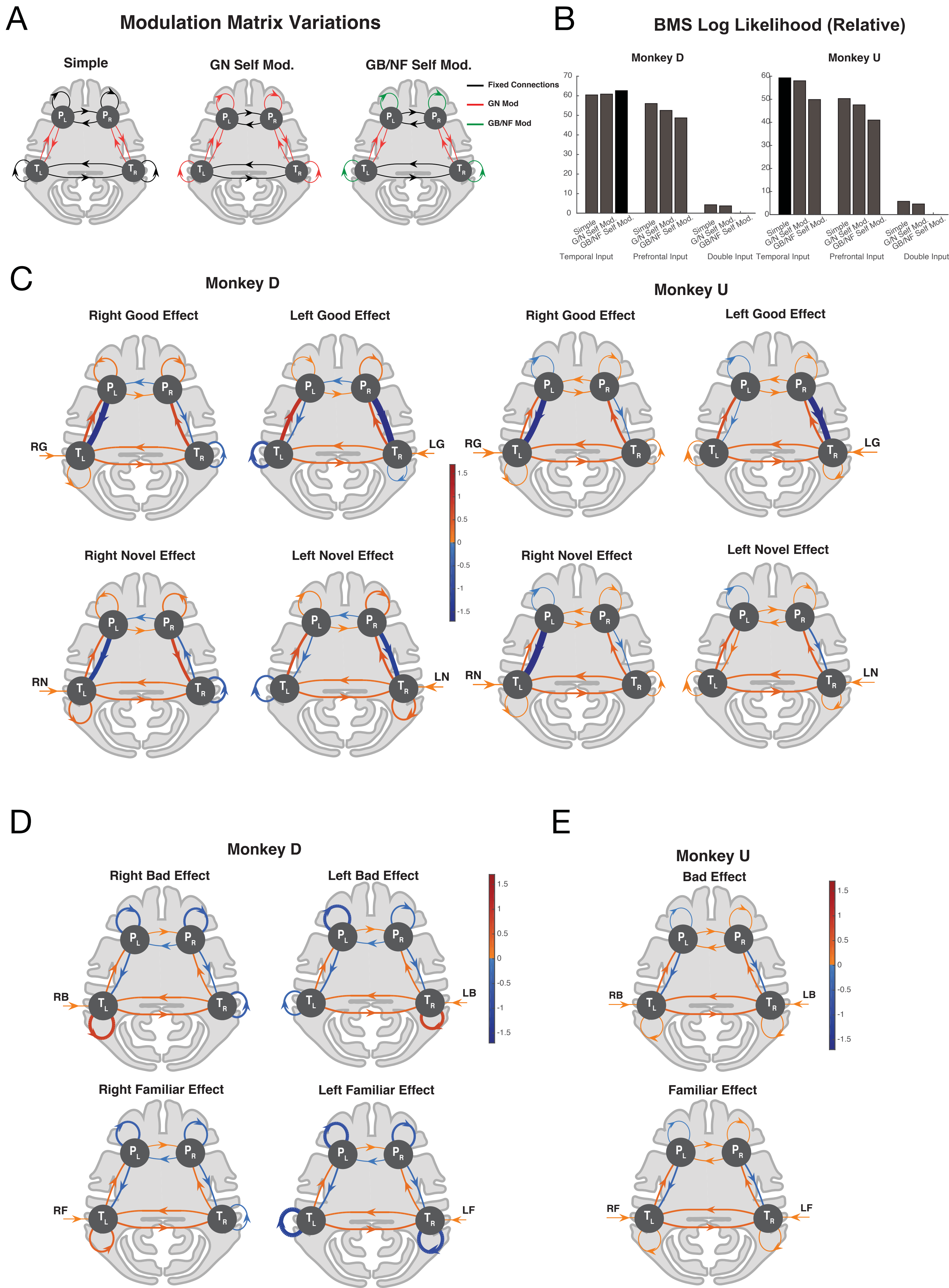
A
Modulation Matrix Variations
Simple
GN Self Mod.
GB/NF Self Mod.
Fixed Connections
GN Mod
GB/NF Mod
B
BMS Log Likelihood (Relative)
Monkey D
Monkey U
70
60
50
40
30
20
10
0
Simple
G/N Self Mod.
GB/NF Self Mod.
Temporal Input
Prefrontal Input
Double Input
C
Monkey D
Monkey U
Right Good Effect
Left Good Effect
Right Novel Effect
Left Novel Effect
RG
LG
RN
LN
1.5
1
0.5
0
-0.5
-1
-1.5
D
Monkey D
Right Bad Effect
Left Bad Effect
Right Familiar Effect
Left Familiar Effect
RB
LB
RF
LF
E
Monkey U
Bad Effect
Familiar Effect

Supplementary Figure 7: The DCM models considered for the TP network and the fitted weights for each object type. A, Three hypotheses regarding the modulation of self-connections. The black lines indicate no modulation. The red lines show edges modulated by the novel and good objects, and the green lines depict edges modulated by all object types: novel, familiar, good and bad. There were also three hypotheses about the input structure: only to temporal, only to prefrontal and to both structures (both) B, Bayesian model selection using relative log likelihood of nine proposed models for each monkey. In both monkeys the input only to temporal had higher likelihood. GBNF self-modulation was best for monkey D and no self- modulation (simple model) was best for monkey U. Best model with the highest posterior is colored black C, DCM model of the prefrontal-temporal interaction during good and novel objects separately for right and left blocks in both monkeys. (TR, TL :right and left temporal nodes, PR, PL :right and left prefrontal nodes). The line widths are scaled by the absolute value of the edges. Signed weights are color encoded. D, same as C but for bad and familiar object presentation for monkey D. E, same as C but for bad and familiar object presentation for monkey U. Since there was no modulation of weights by bad and familiar objects in this monkey both hemifield presentations shared the same weights.

Supp Fig 8

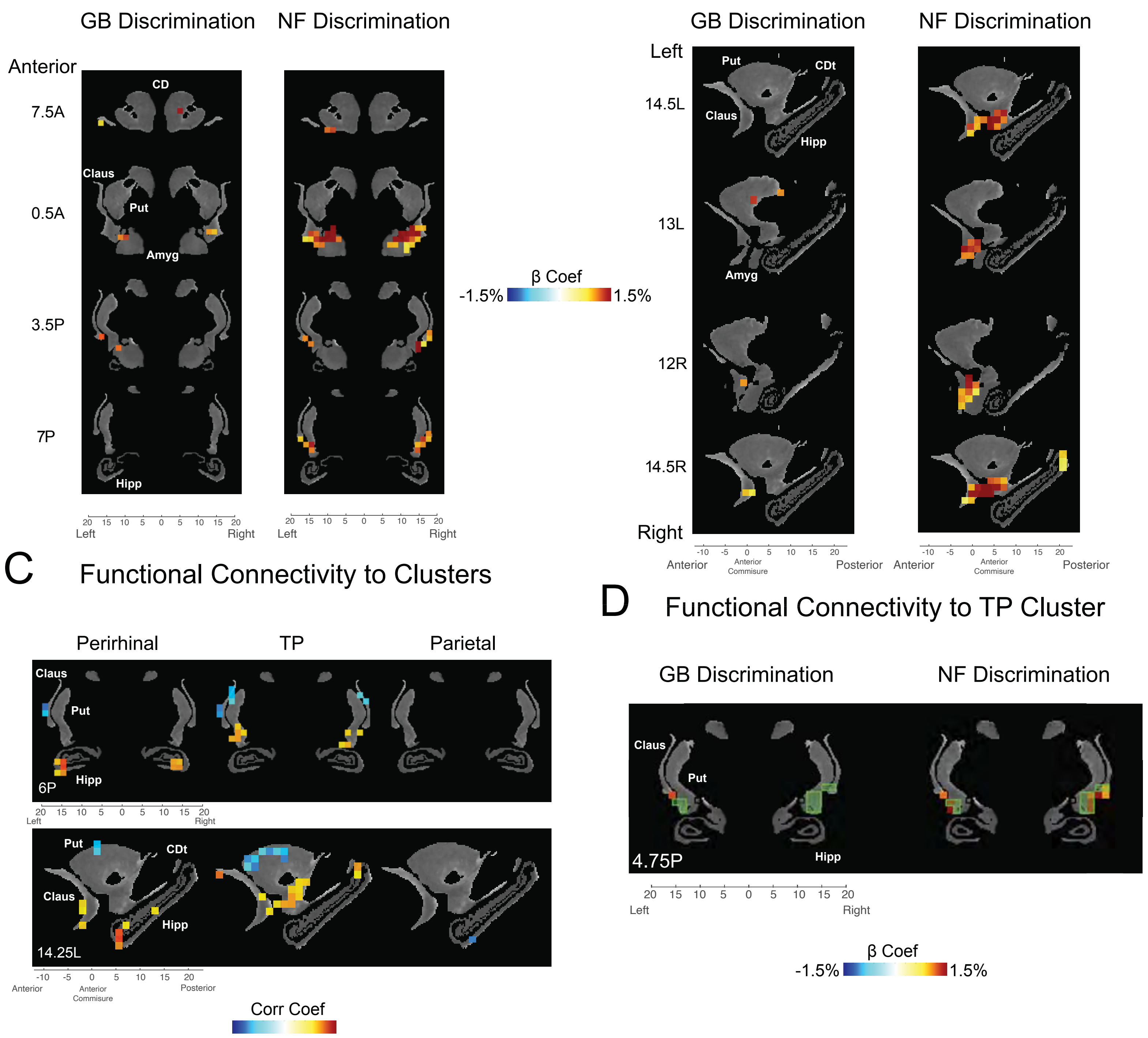

Supplementary Figure 8: Subcortical novelty and value coding in striatum, amygdala, claustrum, and hippocampus. same format as Figure 5 but for monkey D.
